# The Emergence of SARS-CoV-2 Variants With a Lower Antibody Response: A Genomic and Clinical Perspective

**DOI:** 10.3389/fmed.2022.825245

**Published:** 2022-05-06

**Authors:** Suvro Biswas, Shafi Mahmud, Mohasana Akter Mita, Shamima Afrose, Md. Robiul Hasan, Gobindo Kumar Paul, Mst. Sharmin Sultana Shimu, Md. Salah Uddin, Shahriar Zaman, Moon Nyeo Park, Abolghasem Siyadatpanah, Ahmad J. Obaidullah, Md. Abu Saleh, Jesus Simal-Gandara, Bonglee Kim

**Affiliations:** ^1^Miocrobiology Laboratory, Department of Genetic Engineering and Biotechnology, University of Rajshahi, Rajshahi, Bangladesh; ^2^Department of Genetic Engineering and Biotechnology, University of Rajshahi, Rajshahi, Bangladesh; ^3^Department of Pathology, College of Korean Medicine, Kyung Hee University, Seoul, South Korea; ^4^Ferdows School of Paramedical and Health, Birjand University of Medical Sciences, Birjand, Iran; ^5^Drug Exploration and Development Chair (DEDC), Department of Pharmaceutical Chemistry, College of Pharmacy, King Saud University, Riyadh, Saudi Arabia; ^6^Nutrition and Bromatology Group, Department of Analytical Chemistry and Food Science, Faculty of Science, University of Vigo, Ourense, Spain

**Keywords:** SARS-CoV-2, variant of concern, antibody, genomic variation, clinical perspective

## Abstract

The emergence of several novel SARS-CoV-2 variants regarded as variants of concern (VOCs) has exacerbated pathogenic and immunologic prominences, as well as reduced diagnostic sensitivity due to phenotype modification-capable mutations. Furthermore, latent and more virulent strains that have arisen as a result of unique mutations with increased evolutionary potential represent a threat to vaccine effectiveness in terms of incoming and existing variants. As a result, resisting natural immunity, which leads to higher reinfection rates, and avoiding vaccination-induced immunization, which leads to a lack of vaccine effectiveness, has become a crucial problem for public health around the world. This study attempts to review the genomic variation and pandemic impact of emerging variations of concern based on clinical characteristics management and immunization effectiveness. The goal of this study is to gain a better understanding of the link between genome level polymorphism, clinical symptom manifestation, and current vaccination in the instance of VOCs.

## Highlights

- SARS-CoV-2 has evolved many variants as a result of genome-level mutations, worsening the current pandemic situation.
- SARS-CoV-2 variants increase transmissibility, viral virulence, and reduce diagnostic sensitivity.
- The vaccine's efficacy has been brought into question due to the emergence of variants containing a new mutation.
- Vaccine effectiveness and clinical management vary among variants.
- Natural immunity hedging and vaccine-induced immunization evasion have become major public health concerns.

## SARS-CoV-2 GENOME AND MUTATIONS

The largest (amid 26 kb and 32 kb) single-stranded positive-sense RNA genome of SARS-CoV-2 shows low genome stability, with about 1,516 nucleotide-level variations in genome-wide annotations and over 9.8 × 10^−4^ substitutions/site yearly (1–4). The genome or viral transcript of SARS-CoV-2 contains two open reading frames (ORFs) expressing non-structural proteins (NSPs) and four genes encoding structural proteins, namely N (nucleocapsid), M (membrane), E (envelop), and S (spike). ORF1a encodes 11 non-structural proteins (NSP1–11), whereas ORF1b encodes five non-structural proteins (NSP12–16), and ORF8, ORF7b, ORF7a, ORF6, and ORF3a genes encode six accessory proteins, the non-structural proteins being primarily functional proteins (enzymes) that act as a prerequisite for viral replication in tandem with methylation to provoke host responses during infection (5–11).

The viral transcript is notable for a large number of recurrent mutations (>15 occurrences) in the Orf1ab region, namely in three sites (Nsp6, Nsp11, and Nsp13 encoding sites) and one in the spike (S) protein (4, 5, 12, 13). In comparison to the original viral genome, a variant is a virus strain with a considerable phenotypic alteration that exhibits unusual characteristics in terms of virulence, transmissibility, and antigenicity (10, 14). It results from either a complicated combinatorial aberration or an abnormal mutation caused by the combination of three factors, including viral replication errors, recombination between two different viral lineages during coinfection, and the stimulation of host RNA-editing mechanisms. Furthermore, each genetic mutation is incapable of causing significant changes in the essential protein for viral replication and infectivity modification (3, 4, 15–18). The Global Initiative for Sharing Avian Influenza Data (GISAID) managed the global SARS-CoV-2 sequence database by submitting over 1.4 million sequences by May 2021, with a total of 3,913 major representative variants genomes being identified. Additionally, variants of concern (VOCs), which include the Alpha (B.1.1.7 and Q lineages) variant, Beta (B.1.351+B.1.351.2+B.1.351.3) variant, Gamma (P.1 and descendent lineages) variant, Delta (B.1.617.1, B.1.617.2 and AY lineages) variant, the and Omicron (B.1.1529 and BA lineages) variant, predominately emerge from the mutation of the spike gene, where ORF1a region of the genome operates as a pre-eminent NSP mutations site (https://www.gisaid.org/hcov19-variants/). There are two VOIs abbreviated as variants of interest: Lambda (C.37+ C.37.1) and Mu (B.1.621+B.1.621.1), as well as one variant under monitor or VUM, which includes an unidentified (B.1.640 and descendent lineages) variant. However, according to CDC, only omicron and delta variants are considered as the VOCs whereas Alpha, Beta, Gamma, and Mu in conjunction with Eta (B.1.525) variant, Lota (B.1.526) variant, Kappa (B.1.617.1) variant, Epsilon (B.1.427 and B.1.429) variant, Zeta (P.2), and an unknown (B.1.617.3) variant are listed as variants being monitored or VBM (https://www.cdc.gov/coronavirus/2019-ncov/variants/variant-info.html). Of these, kappa is the most recent significant variant evolved from the second COVID-19 wave. (https://www.axios.com/variants-tracker). Our primary emphasis will be the VOCs, VOIs, VUM, or VBMs that play significant roles in SARS-CoV-2-related public health issues (Table 1).

**Table 1:** History and major characteristics of SARS-CoV-2 variants.

**SARS-CoV-2 variant**	**Variant type**	**First identified**	**Major geographic distribution**	**Predominant spike mutations**	**Effects on transmissibility**	**Effects on virulence**	**References**
Alpha	VOC	UK, September 2020	Worldwide	N501Y	Increased	Increased	(10)
Beta	VOC	South Africa, October 2020	Africa	K417N, E484K, N501Y	Increased	Increased	(10)
Gamma	VOC	Japan and Brazil, December 2020	South America	K417T, E484K, N501Y	Increased	Increased	(10)
Delta	VOC	India, December 2020	Worldwide	L452R, E484Q, T478K	Increased	Increased	(10)
Omicron	VOC	South Africa, December 2021	Worldwide	N501Y, K417N, T478K, E484A, D614G	Increased	Reduced	(19, 20); (https://www.gisaid.org/hcov19-variants/); (https://www.cdc.gov/coronavirus/2019-ncov/variants/variant-info.html)
Eta	VOI	United Kingdom/ Nigeria, December 2020	North America	E484K, D614G, Q677H	No evidence	No evidence	(21); (https://www.ecdc.europa.eu/en/covid-19/variants-concern)
Lota	VOI	United States (New York), November 2020	North America	E484K, D614G, A701V, L452R, S477N	No evidence	No evidence	(22); (https://www.ecdc.europa.eu/en/covid-19/variants-concern)
Kappa	VOI	India, December 2020	Asia	L452R, E484Q, D614G, P681R	Increased	No evidence	(23, 24); (https://www.ecdc.europa.eu/en/covid-19/variants-concern)
Epsilon	VOI	California, July 2020	North America	L452R, D614G	Unclear	No evidence	(25); (https://www.ecdc.europa.eu/en/covid-19/variants-concern)
Zeta	VBM	Brazil, January 2021	South America	E484K, D614G	Reduced	No evidence	(21, 26); (https://www.ecdc.europa.eu/en/covid-19/variants-concern)
Lambda	VOI	Peru, August 2020	South America	L452Q, F490S, D614G	Increased	Increased	(27, 28); (https://www.gisaid.org/hcov19-variants/); (https://www.ecdc.europa.eu/en/covid-19/variants-concern)
Mu	VOI	Colombia, January 2021	South America	R346K, E484K, N501Y, D614G, P681H	Increased	No evidence	(29); (https://www.ecdc.europa.eu/en/covid-19/variants-concern)

## Spike Mutations

**Figure 1:**
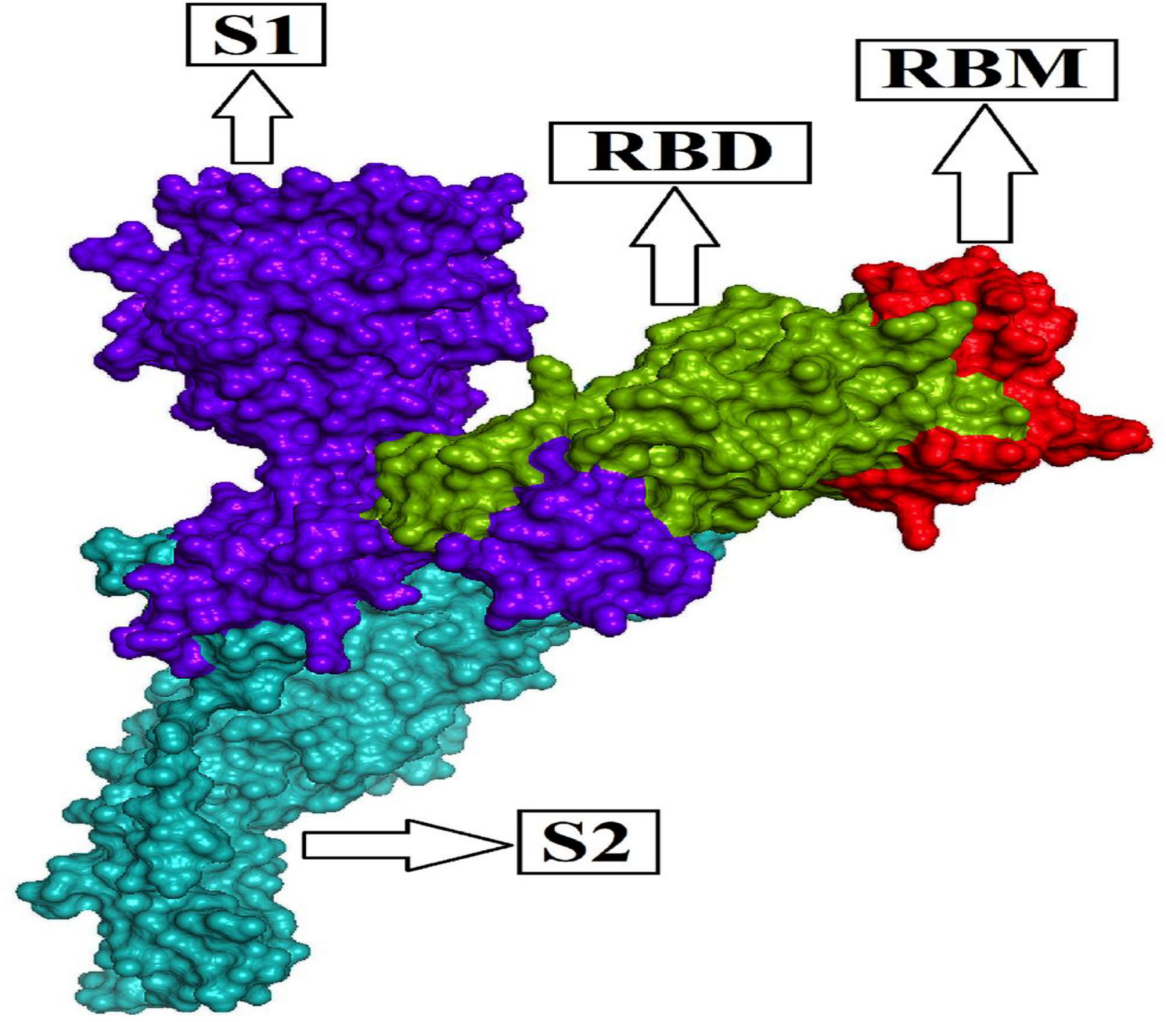
Functional subunits of the spike glycoprotein (S) of SARS-CoV-2.

The homo-trimeric spike protein (S) of SARS-CoV-2 is similar to an obligatory protein that conducts viral entry for virus attachment during infection by recognizing receptors [angiotensin-converting enzyme (ACE2)] in conjunction with cell membrane fusion form. The spike protein is divided into two subunits: S1 subunit comprises the receptor-binding domain (RBD), which can bind to the PD (peptidase domain) of ACE2, and S2 subunit conducts cell membrane fusion *via* the explicit two-heptad repeat region using the six-helical bundle generation (9, 30–33). The RBD's most important operative motif, known as the receptor-binding motif (RBM; Figure 1) evolves the interface between hACE2 and the S protein while maintaining RBD structural stability. As a result, the S1 subunit, which is considered a mutation hotspot with significant clinical relevance, including host immune evasion, transmissibility, and virulence, provides a common key for antibody (Ab) neutralization, as well as future cross-reactive antibody recognition (34–37). The Alpha variant identified in the UK in September 2020 has numerous spike glycoprotein alterations, including K1191N, D1118H, S982A, T716I, P681H, D614G, A570D, N501Y, S494P, E484K, 144del, 70del, 69del, T478I, F490S, E484Q, T478K, T478A, S477N, L455F, Y449S, Y449H, and K417T (https://www.cdc.gov/coronavirus/2019-ncov/variants/variant-info.html); (https://www.gisaid.org/hcov19-mutation-dashboard/).

The N501Y mutation of the alpha variant denotes the substitution of asparagine (N) for tyrosine (Y) at amino acid residue 501; similarly, K417N mutations denote the substitution of lysine (K) for asparagine (N) at amino acid residue 417. However, an Alpha (B.1.1.7) descending evolving variation occupies the E484K mutation, which results in the glutamic acid E being replaced by lysine K at the 484 residues. The Beta variation has the E484K mutation, but the Gamma variant has the K417T mutation in tandem with the E484K mutation, indicating that Beta has numerous substitutions in combination with N501Y, as discovered in South Africa in October 2020 and Brazil/Japan in December 2020 (https://www.gisaid.org/hcov19-variants/). Spike protein substitutions such as A701V, D614G, N501Y, E484K, K417N, 243del, 242del, 241del, D215G, and D80A are provided by the Beta variants, whereas the Gamma variants provide T1027I, H655Y, D614G, N501Y, E484K, K417T, R190S, D138Y, P26S, T20N, and L18F spike protein substitutions (https://www.cdc.gov/coronavirus/2019-ncov/variants/variant-info.html). Delta and Kappa variants, which were first identified in India in December 2020 and are notable as recent influential variants with constant mutations, include E484Q, which refers to the substitution of E (glutamic acid) by Q (glutamine) at the 484 residues, and L452R, which refers to the substitution of L (leucine) by R (arginine) at the 452 residues (https://www.gisaid.org/hcov19-variants/). Additionally, The Delta variant occupies diverse spike glycoprotein substitutions including D950N, P681R, D614G, T478K, L452R, K417N, W258L, A222V, R158G, G142D, T95I, V70F, T19R, G504D, V503F, N501Y, N501T, P499L, S494P, S494L, Q493E, Q493L, F490W, F490L, Y489L, N487T, F486Y, E484Q, E484K, S477C, S477N, S477I, A475T, K458N, L455F, G446V, V445I, and K417T (https://www.cdc.gov/coronavirus/2019-ncov/variants/variant-info.html); (https://www.gisaid.org/hcov19-mutation-dashboard/). The T478K mutation, which refers to the substitution of T (threonine) for K (lysine) at amino acid position 478, is a strange Delta variant mutation.

In addition, numerous spike protein substitutions are notable concerning the Eta (F888L, Q677H, D614G, E484K, 144del, 70del, 69del, and A67V), Lota (Q957R, D950H, T859N, A701V, D614G, E484K, S477N, L452R, D253G, F157S, T95I, D80G, and L5F), and Kappa (Q1071H, T95I, P681R, D614G, E484Q, L452R, E154K, and G142D) variants according to the CDC (https://www.cdc.gov/coronavirus/2019-ncov/variants/variant-info.html). T76I, L452Q, G75V, F490S, D614G, and T859N substitution for the lambda (C.37) variant (37) and S13I, W152C, D614G, and L452R substitution for the epsilon (B.1.427/B.1.429) variant were recognized in the spike gene titled S gene (https://covdb.stanford.edu/page/mutation-viewer/#sec_epsilon). The recent variant named “Omicron” first identified in South Africa has several spike protein substitutions such as del142-144, Y145D, del211, A67V, del69-70, T95I, L212I, ins214EPE, G339D, K417N, N440K, G446S, S371L, S373P, S375F, S477N, T478K, E484A, Q493R, Y505H, T547K, D614G, G496S, Q498R, N501Y, H655Y, N679K, P681H, Q954H, N969K, L981F, N764K, D796Y, and N856K. The omicron mutation N501Y is identical to the mutations mentioned in alpha, beta, gamma, and delta variants. K417N mimics the substitution of alpha and beta variants that differ from gamma and delta variants, and it has the T478K strange delta variant substitution in tandem with E484A substitution that is not observed in any of the above-mentioned-variants, and notable D614G substitution is also present. (https://www.gisaid.org/hcov19-variants/);(https://www.cdc.gov/coronavirus/2019-ncov/variants/variant-info.html). However, on 26 November 2021, the World Health Organization (WHO) designated this “Omicron (B.1.1.529)” as a Variant of Concern due to its high transmissibility and danger of immunological deficiency (38). Table 1 shows the most common spike mutations for SARS-CoV-2 variants.

However, the alterations in the S1 subunit result in a significant increase in S RBD binding affinity for the ACE2 receptor, as well as a decreased affinity for antibody (Ab) neutralization. For example, the B.1 lineage of Beta variants with the D614G mutation in the spike protein shows a 4.3-fold antibody reduction and a 3.5-fold antibody neutralization rebate on average. However, a new Beta variation (501Y.V2), capable of reinfection in COVID-19 convalescent patients, resides in the E484K spike protein mutation, demonstrating the ability to evade first-wave anti-SARS-CoV-2 neutralizing antibodies.

Furthermore, the presence of E484K or N501Y mutations in the S1 subunits causes greater virulence and transmissibility, as well as a higher fatality rate and morbidity (39–42). The indicated D614G mutation at non-RBD sites alters the spike protein structure, leading to monoclonal antibody neutralization and increased SARS-CoV-2 replication *via* virion infectivity enhancement is notable as a prominent spreading mutation extant in more than 99% prevalent variants. Despite not being capable of boosting binding affinity for ACE2 or neutralization sensitivity, the D614G improves infectivity by increasing the amount of functional S protein, as well as improved spike density due to S1 shedding escape and spike integrity shielding. As a result, the presence of the D614G mutation is associated with more agile transmission *in vivo* and increased replication during an *in vitro* investigation (43–47). The L452R mutation, which was discovered in the spike RBM of SARS-CoV-2, allows the virus to escape HLA-A24-restricted cellular immunity, resulting in increased fusogenicity and viral infectivity, as well as increased viral replication (48). Recurrent deletion areas (RDRs) encompassing (90%) four separate regions in the NTD show an Ab-recognizing neutralization domain with increasing deletions remaining in the S1 subunit (N-terminus). Deletions occurring in RDRs is notable in maximum in Alpha-originated variants (e.g., S: ΔHV 69–70, S: ΔY144 in ΔRDR1, and ΔRDR2 respectively), in tandem with Beta-stemmed variants (e.g., S: ΔLAL 242–244, ΔRDR4) and B.1.36 (e.g., S: ΔI210, ΔRDR3), which ends in the resistance for antibody neutralization, wiped epitopes, support in host's immune evasion together with vaccines, or Abs neutralizing declination (10, 49). Furthermore, the top 10 RBD region mutations comprise S494P, T478K, E484K, N501Y, K417N, L452R, K417T, N439K, F490S, and S477N substitutions, observed in 2385, 83587, 19505, 96499, 1129, 83717, 8646, 1930, 499, and 6102 SARs-CoV-2 isolated sequence among 298,376 sample sequences, respectively. Notably, the most prevailing mutations N501Y were detected majorly in the Unites States during April 2021, while L452R and T478K were identified in the United Kingdom during June 2021 (https://www.cbrc.kaust.edu.sa/covmt/index.php?p=top-rbd-variants-heatmap).

**Figure 2:**
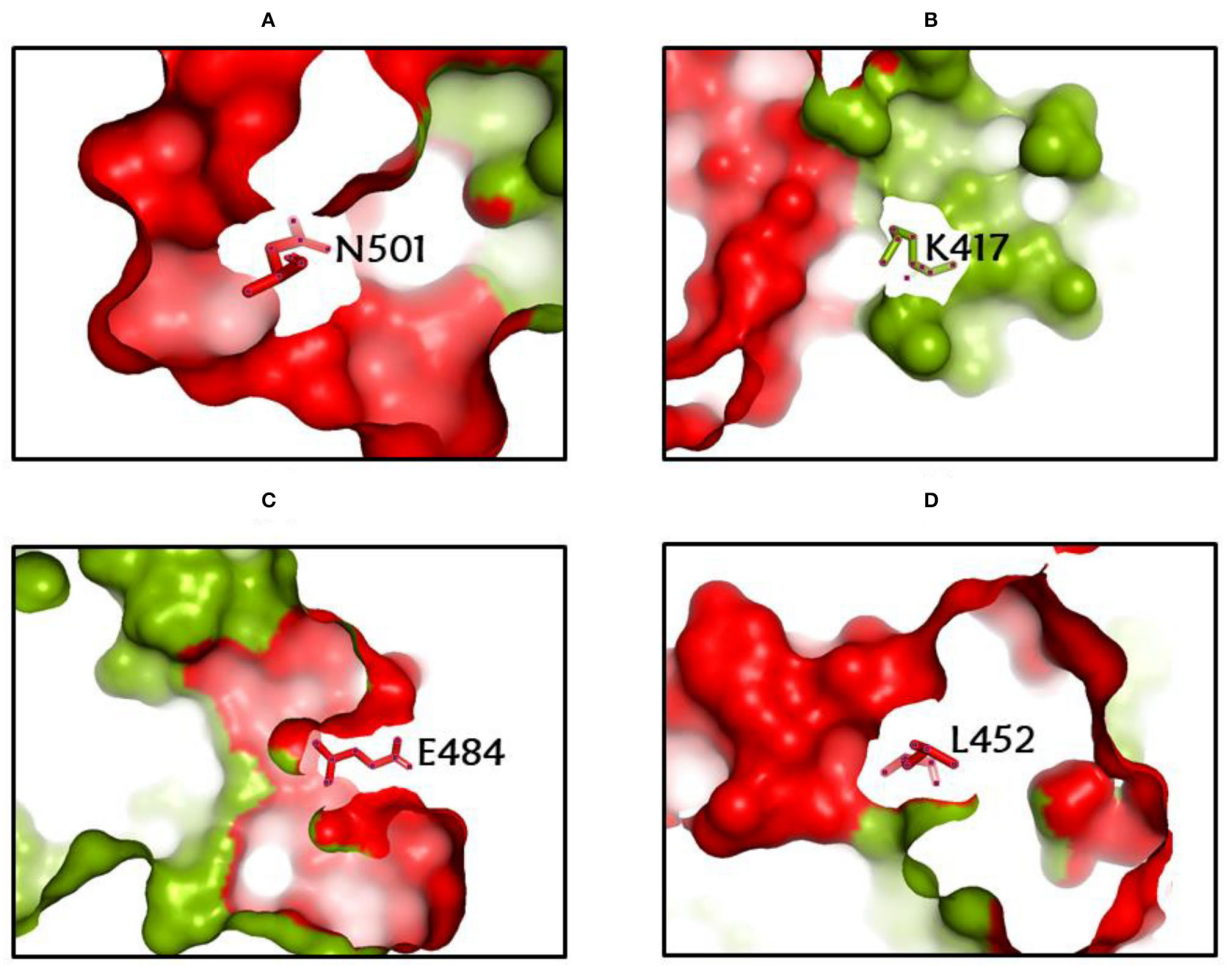
**(A-D)** The most common spike glycoprotein residues (N501, K417, E484, L452), where diverse mutations occur, resulting in different SARS-CoV-2 VOCs, VOIs, and VBMs.

**Figure 3:**
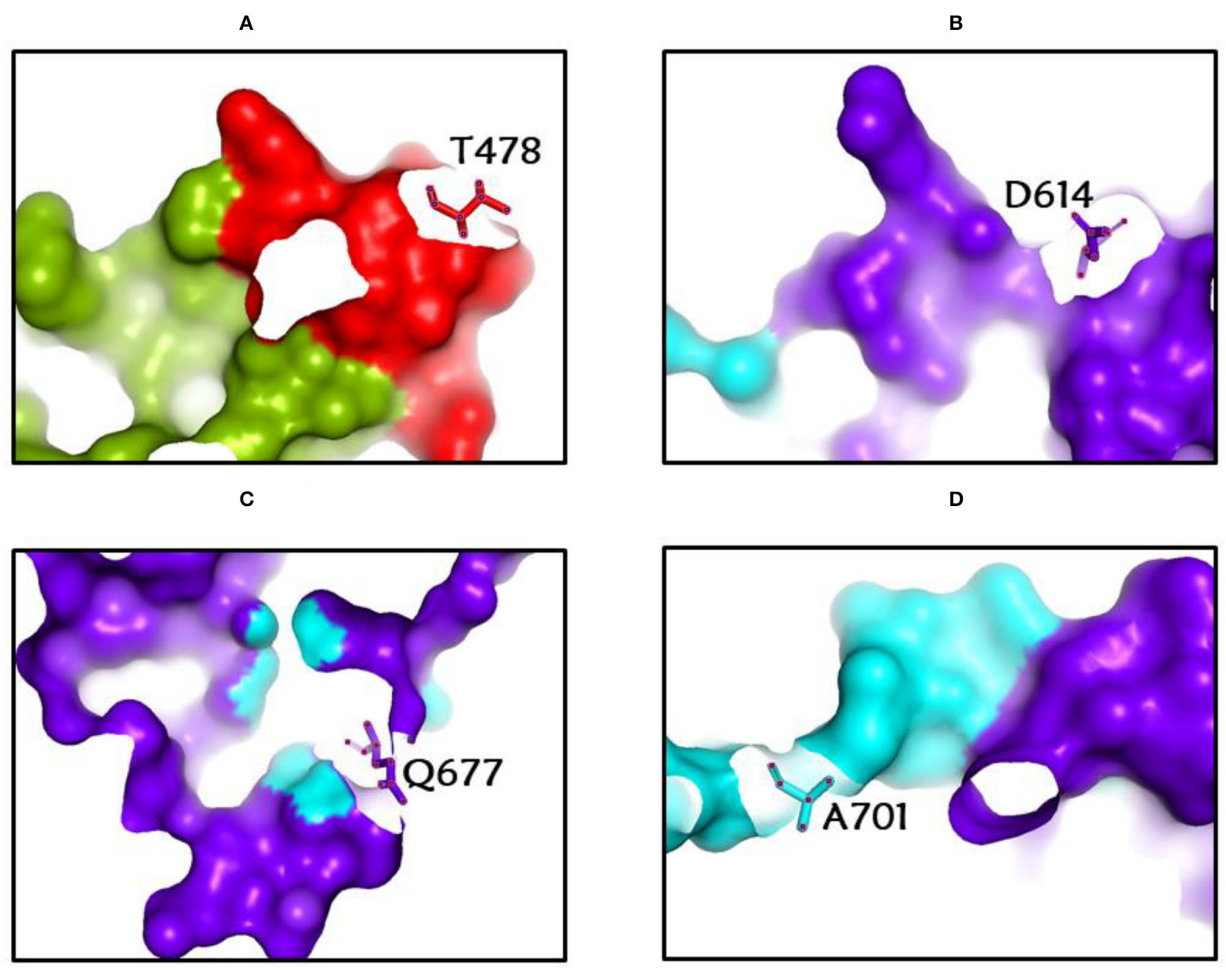
**(A-D)** The most common spike glycoprotein residues (T478, D614, Q677, A701) where diverse mutations occur, resulting in different SARS-CoV-2 VOCs, VOIs, and VBMs.

**Figure 4:**
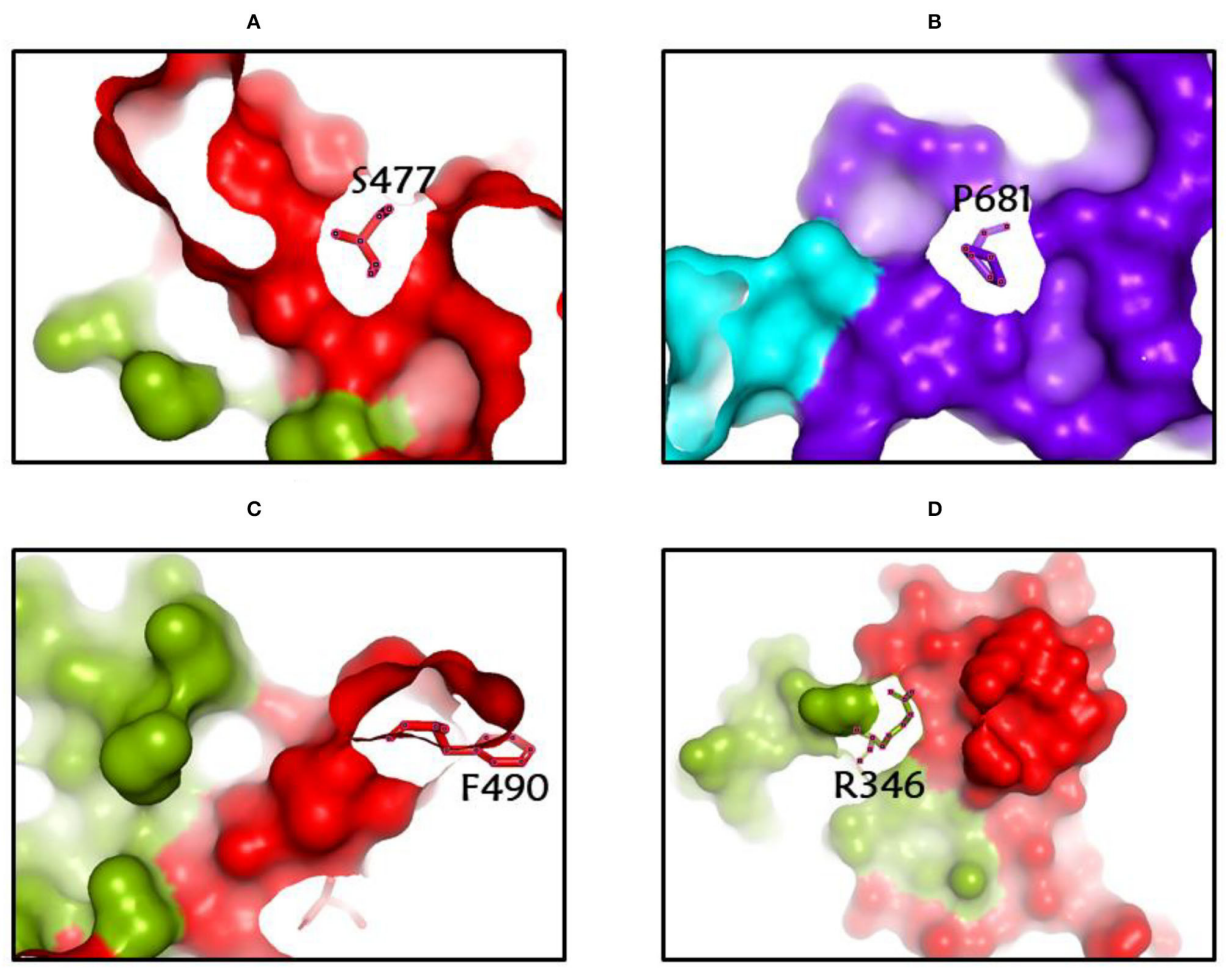
**(A-D)** The most common spike glycoprotein residues (S477, P681, F490, R346) where diverse mutations occur, resulting in different SARS-CoV-2 VOCs, VOIs, and VBMs.

Among the rapidly disseminating arising variants that include alpha, beta, gamma, delta, kappa, eta, lota, epsilon, lambda, mu, and omicron variants, the most well-known D614G mutation (44, 46, 50) provides a reasonable benefit in terms of infectivity (47, 51, 52) and improves transmissibility (53), implying a higher fatality and infectivity rate (54–56). Similarly, the N501Y alteration observed in alpha, beta, gamma, delta, and omicron imparts better ACE2 binding, demonstrating (57–59) the massive increase in ACE2 affinity with a single RBD mutation (57). Furthermore, the E484K mutation in alpha, beta, gamma, delta, kappa, eta, zeta, and lota stimulates escape from multiple mAbs [monoclonal antibodies; (60–62)], as well as antibodies against convalescent plasma (61–63). Again, the K417N/T mutations in the RBD show immune evasion from antibodies and vaccines produced by natural infection (50, 64, 65). Furthermore, both the omicron, delta, and beta variants of K417N and the delta, gamma, and alpha variants of K417T are anticipated to have a lower ACE2-binding affinity (57). Furthermore, the L18F mutation of gamma is responsible for the escape of certain NTD-binding mAbs, resulting in reduced antibody neutralization (66). Similarly, the appearance of the S477N mutation in alpha, delta, lota, and omicron is responsible for resistance to RBD-targeting mAbs-derived neutralization, as well as improved affinity to a lesser extent for the ACE2 receptor. Among the top 10 RBD region mutations, the N439K change improves affinity, but to a lower level in the case of the ACE2 receptor (57, 66). However, an antigenic consequence of the Y144 mutation, which is found in the alpha, eta, and omicron variants, has been observed to prevent neutralization by a number of neutralizing antibodies (66). Furthermore, delta variants with p. 475 (Ala to Val), delta, lota, kappa, and epsilon variants with p. 452 (Leu to Arg), and delta variants with p. 490 (Phe to Leu) increase resistance to a variety of neutralizing antibodies (55). Figures 2–4 show the prominent residues of spike glycoprotein where various mutations occur, resulting in the evolution of different SARS-CoV-2 variants.

## NSP Mutations

ORF8 downregulates host cell major histocompatibility complex class I (MHC-I) in addition to NSP1 positioned in ORF1a/ORF1ab antagonizing activation of type I interferon in host cells, which is associated with SARS-CoV-2 transmissibility and virulence. It is established that the potent suppression of IFN-I signaling causes antiviral activity by combating viral reproduction *via* nsp 1 and nsp 6 in concert with antagonization of the IFN-I response *via* nsp 6, nsp 13, and (ORF6), resulting in the avoidance of the host immune response. The D500-532 mutation in Nsp1 reduces IFN-I response in SARS-CoV-2-infected host cells, as well as during transcription and protein translation in transfected cell lines [HEK293T and A549; (63, 67)]. Additionally, an ill-timed stop codon identified in the Alpha version at position 27 is found in the immune-evasive ORF8 protein, which conducts evasion functions and unique immunological suppression.

However, the most recent major African variants, ORF8: 382 variant and NSP1: 500–532 variant, both of which affect around 5% of global infections and were found in Singapore and China, respectively, contain both ORF8 and NSP1 partial deletions, resulting in lower SARs infection with the CoV-2 virus [https://www.gisaid.org/hcov19-variants/; (68–70)]. Furthermore, 382 SARS-CoV-2 moderate infection variants shorten ORF7b and abolish ORF8 transcription, reducing severe COVID-19-associated proinflammatory cytokines, chemokines, and growth factors (70–72). Important mutations in nsp 2 (T265I), nsp 12 (P4715L), and nsp 13 (P5828L and Y5865C), which serve as helicase or replicase, have also been identified in the United States (73). However, V121D destabilizing NSP-1, G1691C reducing NSP-3 flexibility in combination with V843F, A889V substitution, and V843F dominating substitution in combination with A889V in PLPro has indicated the possibility of an attenuated vaccine in combination with PLPro inhibitors (74, 75).

## PROBABLE CLINICAL IMPACTS OF SARS-CoV-2 VARIANTS

### Increased Transmissibility and Viral Virulence

The Delta variation was linked to a high viral load, high transmission rates, and reinfection (PANGO lineage: B.1.617.2). Unlike the Alpha variant and monoclonal antibodies used in SARS-COV-2 medicines, this version increases rather than decreases susceptibility to the virus (76, 77). In comparison to the Alpha VOC, the Delta (B.1.617.2) VOC was predominantly observed in the younger age group, putting patients at risk of a second hospitalization if they had more than five comorbidities. *In vitro* neutralization experiments using convalescent serum and monoclonal antibodies, and a subject testing of immunized serum show that the Delta variation increases vaccination resistance, especially in individuals with one dosage (76–78).

The Delta SARS-CoV-2 mutant was shown to be 60% more infectious than the wild type and able to evade adaptive immunity in half the time. The S-protein mutation D614G of the Delta variant has been shown to affect virulence and virus transmissibility by preserving a stronger affinity for olfactory epithelium and enhanced virion stability (10, 79). On the other hand, because of their increasing transmissibility and massive mutations in the spike gene, both the alpha variant B.1.1.7 and the beta variant B.1.351 are gaining popularity. It is turbulent for monoclonal antibodies to neutralize the N-terminal region of the spike protein in the B.1.1.7 variant. Evidence suggests that the transmission rate of Alpha and Beta variants of concerns is growing by about 50% in children and younger people (10, 65, 80).

When comparing clinical outcome records, it appears that B.1.1.7 infection has a 30% higher fatality rate than other SARS-CoV-2 variants, which could be attributable to alterations in the receptor-binding domain that make them immune to neutralizing antibodies (10, 81, 82). Furthermore, the B.1.351 variant improves the neutralization of many monoclonal antibodies against the RBD at receptor-binding sites, resulting in an E484K substitution mutation and a 9.4-fold increase in resistance through plasma convalescence (65, 83). Several studies show that the B.1.1.7 variation is around 35–45% more transmissible across the country and gains frequency at a double pace every one and a half weeks (82).

The Epsilon variant, CAL.20C (B.1.427/B.1.429), is characterized by three mutations: L452R mutation in the RBD, and W152C and S13I mutations in the N-terminal domain (NTD). In conjunction with higher viral shedding, these variants increase transmissibility by up to 24% (10, 84, 85). In response to the currently circulating strains, various genome sequences of B.1.427/B.1.429 variants arrayed a rapid increase in viral prevalence, with 50% exceeding the transmissibility rate (85, 86). The Gamma variant P.1 (originating in Brazil) indicated a 20.0% increase in hospitalizations compared with non-VOC patients, and spike mutations may have increased virulence, raising ACE2 rapport, although significant information about viral pathogenicity is currently unavailable in these genotypes (10, 83, 87).

### Decreased Diagnostic Sensitivity

Several studies (13, 88, 89) have described a transitory genetic evolution as a result of the geographical viral transmission of SARS-CoV-2 since the genomic sequence of SARS-CoV-2 became easily accessible. Newly arising variants of concerns can impair the sensitivity of RT-PCR-based identification if a mutation arises in a region where both primers and probes may bind. In the RT-PCR experiment, 79% of the primer binding sites are utilized, but the genome has altered in the meanwhile, with the most important GGG AAC substitution (10, 23). During the expanding SARS-CoV-2 outbreak, novel genetic variants may reduce the specificity and sensitivity of RT-PCR-based detection. Active viral recombination and mutation rates, in particular, might undoubtedly disrupt oligonucleotide annealing and impact sensitivity or inclusiveness. An analysis of genetic variants in the widely distributed SARS-CoV-2 genomes reveals a total of 27 probe- or primer-binding sites with a variant frequency of <1% (89–91).

Diagnostic failures are implicated in the Alpha (B.1.1.7) lineage as considerably higher false-negative results by RTPCRs that target the spike (S) gene. Diagnostic performance was unaffected by the Berlin–Charité technique, with nearly 98% of the sequences being detected using contemporary primers/probe sets because the S protein-producing gene was never used as a target in this procedure. For detection tests, expensive qPCR equipment that relies on signal absences rather than the positive indication for a variant presence is required (92, 93). Sequencing the entire variant's genome for both alpha (B.1.1.7) and beta (B.1.351) variant identification in the next generation sequencing (NGS) approach may result in incorrect or inconclusive detection. Several tests on the Delta B.1.1.7 variant reveal that in the three-target gene of RT-PCR diagnostic assay, where positive cases of SARS-CoV-2 infection are on the rise, this variant exhibits an increase in S-gene target failure rather than positive ORF1ab, N target genes (94–96). An exploratory study of the B.1.1.7. lineage revealed the presence of polymorphisms in the amplified sequences, indicating the discovery of new haplotypes. These haplotypes have a low frequency of single nucleotide polymorphisms (SNPs) that affect oligobinding site areas, hindering accurate identification and resulting in false-negative test findings (93, 97).

In France, a novel variant of concern 202012/01 (VOC) with the deletion of the spike (S) at H69–V70 (H69/V70) location was detected, which is also 43–82% transmissible compared with other SARS-CoV-2 variants. This deletion process is linked to an S-gene target failure in (ORF) 1ab, S, and nucleocapsid (N) gene targets, according to an RT-PCR assay (TaqPath kit) (98). The findings from the TaqPath RT-PCR kit uncovers 0.6% of overall prevalence, indicating a limited variations circulation with H69/V70 during the second wave. Three RBD mutations, Y453F, N501Y, and N439K, have been linked to the H69/V70 gene, which reduces SARS-CoV-2 antibody sensitivity (98, 99). Several laboratories experimented with diagnostic primers or probes alignment with a short viral sequence exhibiting mismatches that led to false-negative results due to a worldwide pandemic emergency (100).

Mutant viruses or genetic diversity were shown to have potential mismatches in the primer or probe binding region of the viral genome, resulting in false-negative results. While a single mismatch has little impact, two or three mismatches reduce technique sensitivity, and having more than three mismatches can result in a complete reaction failure (100, 101). SARS-CoV-2 has newly emerged variants of concern, as well as likely mismatches, indicating the importance of molecular surveillance and providing fresh diagnostic tools for future prevalence. On the other hand, it also provides an identification scheme with high sensitivity and specificity, allowing the CRISPR-based diagnostic tools to be developed (10, 102).

### Potential Influence on Vaccination

Spike protein plays a key role in the pathogenicity of SARS-CoV-2, and vaccines based on targeting this spike protein are being developed (10). Meanwhile, B.1.1.7 (Alpha), B.1.351 (Beta), P.1 (Gamma), and B.1.526 (Delta) forms of SARS-CoV-2 are propagating over the world (103, 104). In this part, we will explore the effectiveness of numerous vaccines against these variants.

### Genetic Vaccines

Pfizer-BioNTech and Moderna developed two anti-SARS-CoV-2 vaccines, “BNT162b2” and “mRNA-1273”, both mRNA-based vaccines that were previously licensed (10). According to several studies, the BNT162b2 vaccination was projected to be 89.5% effective against the Alpha variant and 75.0% effective against the Beta form (105). BNT162b2 was found to be 84% effective against the Gamma version (103), and 88% effective against the Delta variant (106). The mRNA-1273 vaccination was shown to be 94.1 % (107–109) effective for the Alpha variant and 96.4 % effective for the Beta form (110). mRNA-1273, on the other hand, had a lower neutralization rate against Gamma and Delta variants (111). According to multiple studies, the effectiveness of BNT162b2 and mRNA-1273 vaccines against Omicron was only 36% after the second dose but increased to 61% after the third dose (38).

Another study looked into whether using various vaccines as booster doses could increase the immunological response to Omicron. Two doses of BNT162b2 vaccination plus one booster dose of BNT162b2 vaccine, as well as two doses of CoronaVac vaccine plus one booster dose of BNT162b2 vaccine provided protection against Omicron. The vaccine effectiveness of these two groups increases by 95% after a booster dose against this variation (112).

### Adenovirus-Based Vaccines

Adenovirus-based vaccines have been approved for both emergency and routine use (10). Among them, the University of Oxford and AstraZeneca's ChAdOx1 nCoV-19 (chimpanzee adenovirus type Y25 vector) or the AZD1222 vaccine was 74.5% effective against the Alpha variant (106, 113, 114) and 10.4% effective against the Beta variant (115). Though the ChAdOx1 nCoV-19 vaccine has yet to be proved to be effective against the Gamma version, it has shown to be 59.8% effective against the Delta form (116). Another Ad26.COV2.S vaccine which is a recombinant, replication-incompetent human adenovirus type 26 vector encoding full-length and stagnates SARS-CoV-2 spike protein (JANSSEN) was found to have about 86% decreased efficiency against Alpha variant (117, 118) as well as a 64% protection against the Beta variant (119). Furthermore, this vaccination appears to be quite practical against the Gamma variation, although no information on its efficacy against the Delta variant has been released. (https://www.covid19immunitytaskforce.ca/literature-review-effectiveness-of-the-covid-19-vaccines-approved-for-use-in-canada-against-circulating-variants-of-concern/). The Gamaleya Research Institute's Sputnik V vaccine has a high virus-neutralizing efficiency against B.1.351, B.1.617.2, and P.1, as well as other variations (115).

### Subunit Vaccines

The recombinant NVX-CoV2373 (Novavax) vaccine contains prefusion, full-length spike protein with 85.6 % and 51% effectiveness against Alpha and Beta variants, respectively (10, 106, 111). None of the protein-based vaccines, on the other hand, have been approved for widespread use.

### Inactivated Virus-Based Vaccines

BBIBP-CorV, BBV152, and CoronaVac are three inactivated virus-based vaccines that have been approved and are widely used in China, India, and Brazil, respectively (10). Among them, BBIBP-CorV is a vaccine manufactured by Sinopharm (Beijing, China), producing vaccine antisera that are compatible for neutralizing the Beta variant (120). BBV152 (Bharat Biotech, India) is a vaccine that showed efficacy against Alpha and Beta variants and was 652% effective against Delta variant (121, 122), whereas CoronaVac (Sinovac Biotech) is 42% efficient against the Gamma variant (123).

To put an end to this discussion about vaccines, it appears that none of them are effective against all SARS-CoV-2 variants, but the majority of the licensed vaccines are partially effective against the Alpha and Beta types.

## Limitations

According to CDC (https://www.cdc.gov/coronavirus/2019-ncov/variants/variant-info.html), previously called VOCs (epsilon, alpha, beta, and gamma) are now designated as the VBMs in the USA, while alpha, beta, and gamma are still mentioned as VOCs according to GISAID (https://www.gisaid.org/hcov19-variants/) because variants are classified based on their potential impact on critical SARS-CoV-2 countermeasures, including vaccines, treatments, and diagnostics, as they are important for public health. As a result, the viewpoint of variant monitoring is at a conflict with time and research data. Furthermore, new spike protein mutations, in combination with genetic and host predisposition, impact the current vaccination efficacy (124). Furthermore, as new strains appear, the need to reexamine vaccination efficiency by experimenting with *in vivo* reduction of viral infection, as well as antibody quantification obtained from *in vitro* exposure reveals a dearth of understanding about vaccine efficacy (125). Furthermore, as SARS-CoV-2 is constantly mutating, accumulating around one new mutation every 2 weeks in the genome (126), Delta Plus is observed with several new mutations concerning ORF1a (A1146T, A3209V, P1604L, T3750I, and V3718S) evolving from delta variant (127), and RBD-ACE2 system analysis of newly emergent variants such as Omicron revealed 32 mutations in S protein, raising significant concern for its transmissibility. As a result, the shifting variations identified through dynamic research in mutation findings necessitate more investigation for vaccination efficacy and variant tracking (128). As a result, it may be inferred that our understanding of variation mutation and the impact of a variant in conjunction with vaccination efficacy data is evolving, and that, while our analysis depicts the current landscape of variant tracking perfectly, it may alter over time.

## Conclusion

When spike protein mutations are combined with non-structural protein mutations reported in emerging VOCs, VOIs, and VBMs, the clinical relevance of each variance changes. As a result, a potentially devastating global health catastrophe occurs from either a novel variant of concern or a variant of interest that has the potential to worsen the infected individual's clinical status. Furthermore, changes in either the spike protein or the NSP protein has a conceivable impact on vaccination, which is an important problem in vaccine efficacy. As a result of this review, it appears that more vaccine development research is needed to ensure that vaccines are effective against all SARS-CoV-2 variants.

## Author Contributions

SB, SM, MM, SA, MH, JS-G and MSa: study concept and design. SB, SM, MM, SA, MH, GP, AO, and MSa: acquisition of data, analyses, and interpretation of data. SB, SM, MM, SA, MH, AO, and MSh: drafting the manuscript. SZ, MU, MSa, MP, AS, BK, AJO, and JS-G: critical revision of the manuscript for important intellectual content. SZ, MU, BK and MSa: technical or material support. MSa, BK, and JS-G: study supervision. BK: fund acquisition. All authors have read and agreed to the published version of the manuscript.

## Funding

This research was supported by Basic Science Research Program through the National Research Foundation of Korea (NRF) funded by the Ministry of Education (NRF-2020R1I1A2066868), and the National Research Foundation of Korea (NRF) grant funded by the Korea government (MSIT) (No. 2020R1A5A2019413). Funding for open access charge: University of Vigo/CISUG.

## Conflict of Interest

The authors declare that the research was conducted in the absence of any commercial or financial relationships that could be construed as a potential conflict of interest.

## Publisher's Note

All claims expressed in this article are solely those of the authors and do not necessarily represent those of their affiliated organizations, or those of the publisher, the editors and the reviewers. Any product that may be evaluated in this article, or claim that may be made by its manufacturer, is not guaranteed or endorsed by the publisher.
